# Developing Eco-Friendly, High-Performance Soy Protein Plywood Adhesive via Core–Shell Hybridization and Borate Chemistry

**DOI:** 10.3390/ma18051144

**Published:** 2025-03-04

**Authors:** Yi Zhang, Longxiang Sun, Xinyu Li, Ziye Fu, Yang Li, Weisheng Sun, Yawei Sun, Rongfeng Huang, Minghui Guo

**Affiliations:** 1 Key Laboratory of Bio-Based Material Science & Technology, Northeast Forestry University, Ministry of Education, Harbin 150040, China; 2 College of Chemistry and Materials Engineering, Zhejiang A&F University, Hangzhou 311300, China; luckyyizhang@163.com (Y.Z.); lllxy128@163.com (X.L.); fzy2276835351@163.com (Z.F.); bmlwthpl2002@163.com (Y.L.); 18268158266@163.com (W.S.); 3 Treessun Flooring Co., Ltd., Huzhou 313029, China; 15325463456@163.com; 4 Research Institute of Wood Industry, Chinese Academy of Forestry, Beijing 100091, China; 5 North Information Control Research Academy Group Co., Ltd., Nanjing 211100, China; huizhang0303@163.com

**Keywords:** soy protein plywood adhesive, core–shell hybrid, borate chemistry, bonding strength, mildew resistance, flame retardant

## Abstract

Developing eco-friendly, high-performance adhesives is crucial for sustainable industrial applications but remains a significant challenge. Herein, a synergistic strategy combining core–shell hybridization and borate chemistry was employed to fabricate a multifunctional soy protein (SPI) adhesive with excellent adhesion. Specifically, a reactive core–shell hybrid (POSS-U) was synthesized via free-radical polymerization using octavinyl-POSS as the core and urushiol (U) as the shell. Sodium borate (SB) was then added as a crosslinker, along with POSS-U and SPI, to prepare the SPI/POSS-U/SB adhesive. The SPI/POSS-U/SB adhesive exhibited a 100% increase in dry shear strength (2.46 MPa) and a wet shear strength of 0.74 MPa, meeting indoor application standards. Due to the thermal shielding and char formation of POSS and SB, the peak heat release rate of the modified adhesive reduced by 25.4%, revealing excellent flame retardancy. Additionally, the modified adhesive remained mold-free for 144 h due to the antifungal properties of urushiol and boron. This work provides an innovative approach for enhancing protein-based adhesives and contributes to the advancement of multifunctional composite materials.

## 1. Introduction

Adhesives are indispensable in modern industries such as construction, packaging, automotive, and electronics [1,2]. However, most commercially available adhesives are non-renewable, formaldehyde-based resins, which release toxic gases during their production and use, posing significant threats to both the environment and human health [3]. Therefore, developing environmentally friendly and sustainable adhesives to replace formaldehyde-based resins has become a critical task [4,5,6].

Soy protein adhesives are particularly promising alternatives to formaldehyde-based resins, owing to their sustainability, environmental protection, and low cost [7,8,9]. Nevertheless, the large-scale application of soy protein adhesives is hindered by inherent challenges, such as insufficient water-resistant adhesion strength and limited functionality [10,11,12,13]. To address these deficiencies, researchers have explored various modification strategies [14,15,16,17]. Among these, crosslinking was the most effective method for enhancing the adhesion performance by reacting with the functional groups (e.g., -NH_2_, -OH, and -COOH) of soy protein to form dense, crosslinked networks [18,19]. However, a large amount of crosslinker is often required to achieve sufficient adhesion strength due to the low reactivity of soy protein [20], and most crosslinkers are derived from petroleum-based resources, which increases the reliance of the soy protein adhesive on non-renewable resources. Furthermore, the high-density crosslinked structure formed can exacerbate the brittleness of the adhesive layer, leading to brittle fractures in bonded products [21,22]. Moreover, the cross-linking modified soy protein adhesives often lack additional functionalities, such as flame retardancy and anti-mold properties [23]. Therefore, current soy protein adhesives still face challenges related to low bonding strength, poor water resistance, high brittleness, and limited functionality, highlighting the need for innovative modification strategies.

Core–shell compounds, as an emerging class of hybrid materials, have shown the potential to enhance strength without compromising the toughness of composites [24,25,26]. For instance, Li et al. [27] reported a core–shell particle synthesized by grafting amino-terminated hyperbranched polymer onto silica nanoparticles. Adding 3 wt% of the core–shell hybrid significantly improved both the tensile strength and impact toughness of epoxy resin. In core–shell hybrids, the high-modulus nanoparticles act as core materials, which enhances the mechanical strength of the composites. Additionally, under external impact forces, the nanocores act as physical barriers to induce crack deflection, thereby improving the toughness of composites. Moreover, by selecting different types of core materials, multifunctionalities such as flame retardancy, antimicrobial activity, and electrical conductivity can be achieved. For example, the polyhedral oligomeric silsesquioxane (POSS) is an ideal core material for core–shell hybrids, since it consists of a cage-like inorganic silicon–oxygen framework that restricts molecular chain motion, imparting superior mechanical properties, thermal stability, and flame retardancy to hybrid materials [28,29]. To improve interfacial compatibility between nanocores and organic matrices, our previous studies demonstrated that natural polyphenols (e.g., tannin, urushiol) with catechol groups can mimic the strong adhesion of mussel proteins, forming covalent and non-covalent interactions between the organic matrix and inorganic particles [30]. Thus, constructing reactive core–shell structures using POSS as the core and plant polyphenols as the reactive shell is expected to enhance the adhesion performance and multifunctionality of soy protein adhesive. However, in practice, achieving economic feasibility while relying solely on the addition of low-dosage core–shell materials composed of nanomaterials and polyphenols is insufficient to form an efficient three-dimensional crosslinked network within the adhesive matrix.

Interestingly, in higher plant cell walls, extremely low concentrations of borates can efficiently crosslink pectic polysaccharides containing multi-hydroxyl groups. This crosslinking stabilizes the cell wall structure, effectively enhancing its mechanical properties [31]. The high crosslinking efficiency of borates is attributed to the unique chemical properties of the boron atom, which not only coordinates with single hydroxyl groups over a wide pH range but also preferentially reacts with two diols to form stable borate diester bonds [32]. These bonds connect multiple polymer chains, constructing a three-dimensional crosslinked network. Additionally, boron exhibits low toxicity, flame retardancy, antimicrobial activity, and anti-mold properties. Inspired by the borate chemistry in plant cell walls, researchers have developed a range of advanced materials [33]. Building upon this inspiration, the incorporation of environmentally friendly borates in synergy with POSS-polyphenol core–shell structures is expected to simultaneously enhance the adhesive strength and toughness of soy protein adhesives. This approach not only preserves the environmental benefits of bio-based adhesives but also imparts multifunctionality.

In this study, we aimed to develop a high-performance, environmentally friendly, and multifunctional bio-based soy protein adhesive by leveraging core–shell hybridization and borate chemistry inspired by plant cell walls. Specifically, we designed and synthesized a reactive core–shell hybrid (POSS-U) using octavinyl-polyhedral oligomeric silsesquioxane (octavinyl-POSS) as the core and urushiol (U) as the shell through free radical polymerization. Subsequently, sodium borate (SB), as an efficient borate-based crosslinker, was incorporated alongside POSS-U into the soy protein isolate (SPI) to fabricate a SPI/POSS-U/SB adhesive. This innovative strategy provides valuable insights for developing advanced plant protein-based adhesives and is expected to drive technological innovation in the field of multifunctional composites.

## 2. Materials and Methods

### 2.1. Materials

Soy protein isolate (SPI) with 95% protein content was purchased from Shandong Yuxin Bio-tech Co., Ltd. (Binzhou, China). Raw lacquer, sodium borate (SB), ammonium persulfate (APS), sodium hydroxide (NaOH), and other reagents were purchased from Shanghai Macklin Biochemical Technology Co., Ltd. (Shanghai, China). Octavinyl-POSS was purchased from Aladdin Biochemical Technology Co., Ltd. (Shanghai, China). Poplar veneers (*Populus tomentosa Carrière*, 40 cm × 40 cm × 1.5 cm, 8% of moisture content) were purchased from the Shandong Linyi Wood Panel Factory (Linyi, China).

### 2.2. Extraction of Urushiol (U) from Raw Lacquer

A total of 66.67 g of raw lacquer was combined with 2000 mL of ethanol (ratio: 1 g lacquer to 30 mL ethanol) in a glass beaker and stirred using a magnetic stirrer at room temperature until the raw lacquer was evenly dispersed in the ethanol solution. The resulting dispersion was filtered through a cloth funnel lined with two layers of filter paper. This filtration process was repeated twice to obtain a clarified solution. The ethanol was subsequently removed from the solution using a rotary evaporator (Hei-VAP Core ML G3, Heidolph Instruments GmbH & CO. KG, Schwabach, Germany) at 45 °C and 100 rpm. The resulting brown viscous liquid, obtained after solvent evaporation, was urushiol (referred to as U).

### 2.3. Synthesis of the POSS-U Core–Shell Hybrid

A mixture of 9 g of urushiol (U) and 6 g of octavinyl-POSS was placed in a beaker and stirred evenly using a magnetic stirrer. Subsequently, 0.15 g of ammonium persulfate (APS, representing 1% of the total weight) was added to the mixture and stirred for 5 min at room temperature. The resultant product was the POSS-U core–shell hybrid.

### 2.4. Preparation of SPI/POSS-U/SB Adhesives

The control SPI adhesive, designated as adhesive 0, was prepared by mixing 12 g of SPI with 88 g of deionized water at room temperature for 10 min to achieve a homogeneous dispersion. The modified SPI adhesives containing POSS-U and SB, designated as SPI/POSS-U/SB-n (n = 0.5, 1.5, and 2.5), were prepared as follows: 88 g of deionized water and 0.7 g of sodium dodecyl sulfate (SDS) were mixed, and the pH was adjusted to 9 using 18% NaOH solution. Subsequently, 3 g of POSS-U, 12 g of SPI, and various amounts of SB (0.5, 1.5, and 2.5 g) were added, followed by stirring at 400 rpm using a magnetic stirrer until the mixture was homogeneous. The formulations of all adhesives are detailed in Table 1.

### 2.5. Plywood Preparation and Evaluation

To evaluate the performance of the adhesives, three-ply poplar plywood was prepared using the synthetic SPI adhesives. The adhesive was applied to both sides of the core veneer using a roller coating method, with a coating density of 180 g/m^2^ per surface. The veneers were assembled in the vertical grain direction and pressed under a hot-pressing process conducted at 120 °C, with a pressure of 1.0 MPa for 6 min [34].

After cooling at room temperature for 12 h, the plywood samples were cut into six small specimens (25 mm × 100 mm each). These specimens were soaked in hot water (63 ± 3 °C) for 3 h, removed, and air-dried at room temperature for 10 min. The shear strength and wood failure rate of the plywood were determined using a universal testing machine (5967, Instron, Inc., Norwood, MA, USA) in accordance with the Chinese National Standard (GB/T 17657-2022) [35]. The principle of shear measurement involves applying a tensile load to the plywood specimen, inducing shear failure within the adhesive layer to assess the bonding quality of the plywood. The loading rate during testing was set to 10.0 mm/min [36].

The dry and wet shear strengths were calculated using Equation (1). Each plywood sample was tested for shear strength in six replicates, and the average value was reported.

(1) $$\mathrm{Dry}/\mathrm{Wet}\;\mathrm{shear}\;\mathrm{strength}\;\left(\mathrm{MPa}\right)=\frac{\mathrm{Tension}\;\mathrm{Force}\left(N\right)}{\mathrm{Gluing}\;\mathrm{Area}\;\left(m^{2}\right)}$$

### 2.6. Characterization

#### 2.6.1. Fourier-Transform Infrared (FTIR) Spectroscopy

The U, octavinyl-POSS, POSS-U, and modified SPI adhesives were fully cured, mixed with dried potassium bromide (KBr) powder, and ground to 200 mesh. FTIR spectra of the samples were recorded in the range of 400–4000 cm^−1^ using a Thermo Scientific Nicolet iS20 FTIR spectrometer (Thermo Fisher Scientific, Waltham, MA, USA). Each sample was scanned 32 times at a resolution of 4 cm^−1^.

#### 2.6.2. Flame Retardancy Testing

Flame retardancy was evaluated using a cone calorimeter (CC, FTT, London, UK) following the GB/T 16172-2007 standard [37]. Adhesive samples (3 × 100 × 100 mm) were prepared and tested in a horizontal position under a heat flux of 50 kW/m^2^ [38].

#### 2.6.3. Thermogravimetric (TG) Measurement

The thermal stability of the cured adhesives was analyzed using a TA Q50 thermogravimetric analyzer (Waters, Milford, CT, USA). Approximately 6 mg of each sample was heated from 25 °C to 600 °C at a rate of 10 °C/min under a nitrogen atmosphere, and the weight loss was recorded.

#### 2.6.4. Scanning Electron Microscopy (SEM)

Adhesive samples were evenly coated onto aluminum foil and baked at 120 ± 2 °C until fully dried. The fracture microstructures of the adhesives were observed using a Hitachi SU 8010 cold-field emission SEM (Hitachi Limited, Tokyo, Japan). Samples were gold-coated prior to imaging.

#### 2.6.5. Toughness Evaluation

Toughness evaluation was carried out based on the method reported by Luo et al. [39]. Each sample was spread uniformly on a glass slide to a thickness of ~1.5 mm and dried in an oven at 120 °C for 2 h. Crack propagation on the adhesive surface was photographed at room temperature using a digital camera.

#### 2.6.6. Residual Rate Test

The residual rate was conducted in accordance with the ASTM D5570/D5570M-10(2022) standard [40], which is a key indicator for evaluating the water resistance of adhesives. Adhesive samples were dried in an oven at 120 °C to a constant weight (M_1_), immersed in water at 63 °C for 6 h, then re-dried at 105 °C until a constant weight (M_2_) was achieved. The residual rate was calculated using Equation (2). Six replicates per adhesive formulation were performed, and the average values were reported.

(2) $$\mathrm{Residual}\;\mathrm{rate}\;\mathrm{value}\;\left(\%\right)=\frac{M_{2}}{M_{1}}\times 100\%$$

#### 2.6.7. Mildew Resistance Testing

The mildew resistance test was performed in accordance with the Chinese National Standard (GB/T 1741-2020) [41]. SPI adhesives (Adhesives 0–3) were evenly spread in 80 mm petri dishes and stored in a conditioned environment at 30 ± 2 °C with 80% relative humidity (RH). The mildew resistance test for each adhesive formulation was repeated three times. The samples were monitored daily for visible mildew, and their conditions were recorded with photographs.

### 2.7. Statistical Analysis

All experiments were performed in six replicates, with data presented as the mean ± standard deviation. Standard deviations were represented as error bars in all figures. Statistical analyses were conducted using Statistical Product and Service Solutions (SPSS, Version 20.0) software. Differences were considered statistically significant when the *p*-values were * *p* < 0.05, ** *p* < 0.01, and *** *p* < 0.001.

## 3. Results and Discussion

### 3.1. Synthesis Mechanism of Core–Shell POSS-U

**Figure 1:**
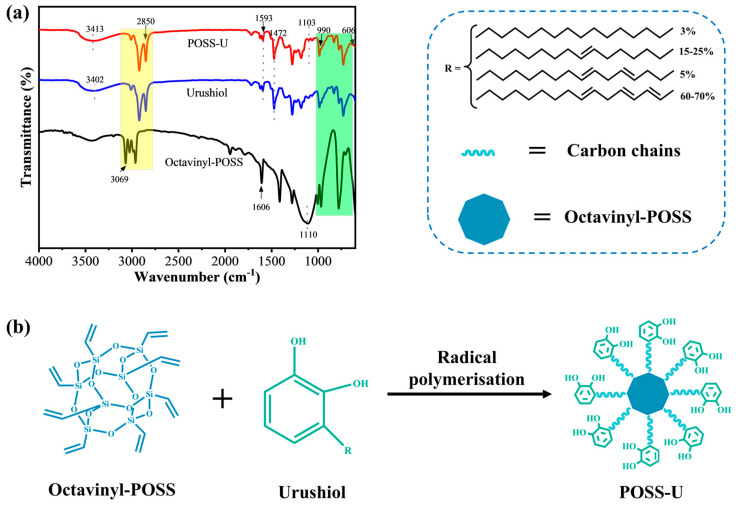
(**a**) FTIR spectra of U, octavinyl-POSS, and POSS-U. (**b**) Schematic representation of the synthesis of core–shell POSS-U.

To investigate the synthesis mechanism of core–shell POSS-U, FTIR analysis was performed, as shown in Figure 1a. In the FTIR spectrum of octavinyl-POSS, a characteristic Si-O-Si peak appeared at 1110 cm^−1^, while the peak at 1606 cm^−1^ was attributed to the C=C stretching vibration. For urushiol (U), a characteristic peak at 3398 cm^−1^ corresponded to the phenolic hydroxyl groups. The peaks at 2920 cm^−1^ and 2850 cm^−1^ were assigned to the stretching vibrations of the -CH_2_ groups in the U side chain, and the C-H bending vibration of the benzene ring in U was observed at 1472 cm^−1^ [42]. The C=C stretching vibration peak of U appeared at 1593 cm^−1^. After the free radical polymerization of octavinyl-POSS and U, the characteristic C-H peak of the benzene ring appeared at 1472 cm^−1^ in the spectrum of the resulting POSS-U. Compared to the octavinyl-POSS spectrum, the POSS-U spectrum exhibited new peaks at 2920 cm^−1^ and 2850 cm^−1^, corresponding to the -CH_2_ stretching vibrations. Additionally, the Si-O-Si stretching vibration peak was observed at 1103 cm^−1^ in the POSS-U spectrum. These results indicated that octavinyl-POSS and U underwent a free radical polymerization reaction via the C=C double bonds to form a core–shell hybrid (POSS-U) with U encapsulating octavinyl-POSS [43]. The synthesis mechanism of POSS-U is depicted in Figure 1b.

### 3.2. Chemical and Microstructural Analysis of the Adhesives

**Figure 2:**
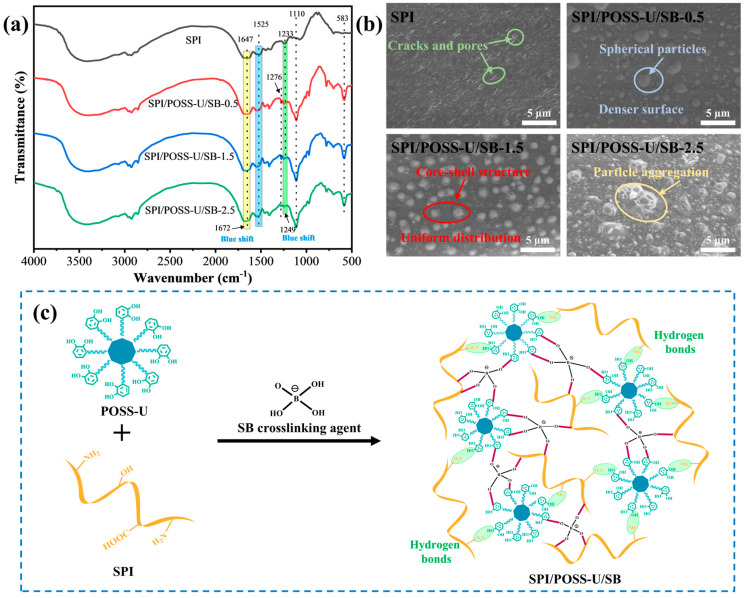
(**a**) FTIR spectra of different adhesive samples. (**b**) Fracture surfaces of different adhesive samples. (**c**) Crosslinking mechanism of SPI/POSS-U/SB adhesives.

To evaluate the effect of the core–shell POSS-U and sodium borate (SB) crosslinking modification on the chemical structure of the SPI adhesive, FTIR spectra were recorded, as shown in Figure 2a. In the FTIR spectrum of pure SPI, a broad peak at 3458 cm^−1^ was attributed to the hydroxyl group of soy protein. Three characteristic peaks at 1647 cm^−1^, 1525 cm^−1^, and 1233 cm^−1^ were assigned to the C=O stretching vibration (amide I), N-H bending vibration (amide II), and N-H/C-N stretching vibration (amide III), respectively. Compared to the pure SPI spectrum, the broad peak in the SPI/POSS-U/SB adhesive shifted from 3458 cm^−1^ to 3438 cm^−1^. This significant red shift was due to the formation of hydrogen bonds between the phenolic hydroxyl groups in the POSS-U shell and the amino/hydroxyl groups in SPI. Furthermore, two new characteristic peaks at 1110 cm^−1^ and 583 cm^−1^ appeared in the SPI/POSS-U/SB adhesive spectrum, which corresponded to the Si-O-Si stretching and bending vibrations in the octavinyl-POSS. These results suggest that the core–shell POSS-U exhibited good interfacial compatibility with the SPI matrix. After the introduction of the borate crosslinking agent (SB), a new B-O bond peak was observed at 1276 cm^−1^ in the SPI/POSS-U/SB adhesive spectrum. This confirmed that SB reacted with the hydroxyl groups in SPI and POSS-U to form borate ester bonds, thus generating a densely crosslinked network. The reaction mechanism of the SPI/POSS-U/SB adhesive is shown in Figure 2c.

The microstructure of the SPI adhesive before and after modification is presented in Figure 2b. The fracture surface of the pure SPI adhesive exhibited numerous cracks and pores, which can be attributed to its inherently loose and porous structure. This porous morphology makes the SPI adhesive susceptible to water penetration, compromising its water resistance. In contrast, after the introduction of POSS-U and SB, the fracture surface of the SPI/POSS-U/SB adhesives became significantly denser, with a notable reduction in pore size and number. Notably, spherical particles were observed on the cross-section of the SPI/POSS-U/SB-0.5 adhesive. In the SEM image of the SPI/POSS-U/SB-1.5 adhesive, these spherical particles were more clearly discernible, revealing a core–shell structure consisting of a central core encapsulated by an outer shell, which could be attributed to the incorporation of the POSS-U hybrid. Moreover, as the SB content increased from 0.5 g to 1.5 g, the distribution of the core–shell POSS-U within the adhesive matrix became more uniform. This was due to the SB acting as a bridge, which enhanced the compatibility between POSS-U and the SPI matrix through borate ester crosslinking, thereby ensuring the sufficient toughness and bonding strength of the adhesive layer. However, when the SB content was further increased to 2.5 g, aggregation of POSS-U particles occurred in the SPI/POSS-U/SB-2.5 adhesive layer, inducing microcracks. This phenomenon could be attributed to the excessive crosslinking density, which increased interactions between POSS-U molecules, leading to their aggregation and compromising the uniformity of the adhesive structure.

### 3.3. Mechanical Properties of Adhesives

**Figure 3:**
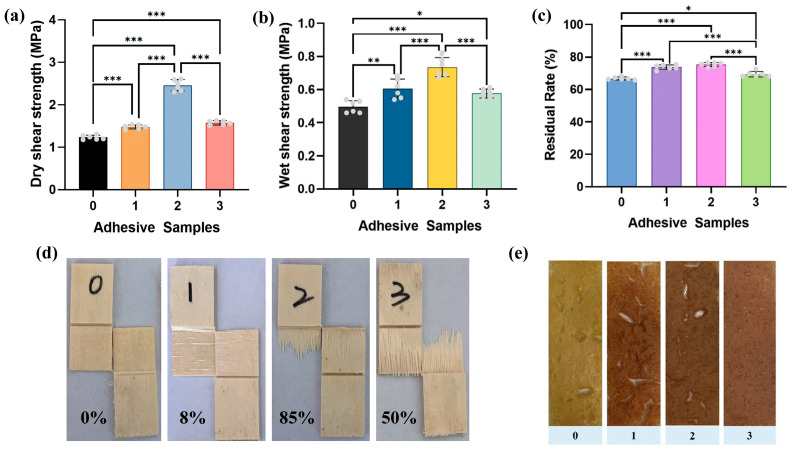
(**a**) Dry shear strength, (**b**) wet shear strength, (**c**) residual rates of different adhesive samples, (**d**) wood failure percentage, and (**e**) crack observations of different adhesive samples: 0 (SPI), 1 (SPI/POSS-U/SB-0.5), 2 (SPI/POSS-U/SB-1.5), and 3 (SPI/POSS-U/SB-2.5). Statistically significant differences were denoted as follows: * *p* < 0.05, ** *p* < 0.01, and *** *p* < 0.001.

To evaluate the adhesion performance, plywood samples bonded with the adhesives were prepared and tested (Figure 3a). The wet shear strength of the pure SPI adhesive was 0.5 MPa, failing to meet the standard for indoor use (≥0.7 MPa). This poor water resistance is due to the weak bonding mechanisms in SPI adhesives, which rely primarily on physical entanglement and hydrogen bonding. The loosely crosslinked structure of pure SPI is highly susceptible to water-induced structure failure. Upon the addition of POSS-U and SB, the wet shear strength of the SPI/POSS-U/SB adhesives improved significantly. Specifically, the SPI/POSS-U/SB-1.5 adhesive achieved a wet shear strength of 0.74 MPa, which was 48% higher than that of pure SPI and met the indoor application standard. Statistical analyses were performed to evaluate the significance of the shear strength differences. As shown in Figure 3a, the results exhibited a highly significant difference in wet shear strength between the SPI/POSS-U/SB-1.5 adhesive and the pristine SPI adhesive (*** *p* < 0.001). Additionally, the dry shear strength of the SPI/POSS-U/SB-1.5 adhesive reached 2.46 MPa, a 100% increase compared to pure SPI (1.23 MPa). Statistical analysis also indicated an extremely significant difference in dry shear strength between the SPI/POSS-U/SB adhesives and the pristine SPI adhesive (*** *p* < 0.001). The improvement in bonding performance could be attributed to the organic–inorganic hybridization effect of core–shell POSS-U, which enhanced the mechanical strength of the adhesive. Simultaneously, SB reacted with the hydroxyl groups of SPI and POSS-U, forming a compact borate ester crosslinked network that synergistically enhanced the bonding strength of the SPI/POSS-U/SB adhesives. However, when the SB content increased to 2.5 g, the bonding strength of the SPI/POSS-U/SB-2.5 adhesive decreased. This could be explained by the aggregation of POSS-U particles caused by the excessive SB content, as observed in the SEM analysis, which compromised the adhesive’s structural stability.

To assess the water resistance of the adhesives, the residual rates were measured (Figure 3b). The pure SPI adhesive exhibited the lowest residual rate (66.67%) due to its low crosslinking density, which allowed unreacted small molecules to hydrolyze during water immersion. After the addition of POSS-U and SB, the residual rate increased, following a trend consistent with the shear strength results. The SPI/POSS-U/SB-1.5 adhesive exhibited the highest residual rate (75.33%), representing a 13% improvement over pure SPI. Statistical analysis revealed an extremely significant difference in residual rate between the modified SPI adhesives and the pristine SPI adhesive (*** *p* < 0.001). This enhancement in residual rate could be attributed to the increased crosslinking density achieved through borate coordination, which formed a dense adhesive structure that effectively prevented water intrusion.

The wood failure rates of the plywood samples subjected to the dry shear strength test were evaluated following the LY/T 2720-2016 standard [44] (Figure 3c). As shown in Figure 3d, the wood failure rate for the plywood bonded with pure SPI adhesive was 0%. In contrast, the modified adhesives demonstrated a significant increase in the wood failure rate. Notably, the SPI/POSS-U/SB-1.5 adhesive achieved a wood failure rate of 85%, indicating that the bonding strength of the SPI/POSS-U/SB adhesive exceeded the inherent strength of the wood itself. This result further confirmed that borate ester crosslinking and core–shell hybridization effectively enhanced the bonding performance of the SPI adhesive.

To visually assess the toughness of the adhesive layers, the adhesives were coated onto glass slides, and their surface morphologies were observed after curing (Figure 3e). The pure SPI adhesive and SPI/POSS-U/SB-0.5 adhesive exhibited numerous cracks and holes after curing, owing to the inherently loose and porous structure of SPI. With increasing SB content, the number of cracks and holes in the cured SPI/POSS-U/SB adhesives progressively decreased. Specifically, the cured SPI/POSS-U/SB-2.5 adhesive layer appeared more compact and flatter, suggesting improved toughness. This enhancement could be attributed to the uniform dispersion of core–shell POSS-U facilitated by the addition of SB, which promoted the formation of an organic–inorganic hybrid network in the SPI/POSS-U/SB adhesive. This network effectively mitigated crack propagation and relieved stress concentration during the curing process, thereby enhancing the toughness of the adhesive.

### 3.4. Flame-Retardant Properties of Adhesives

**Figure 4:**
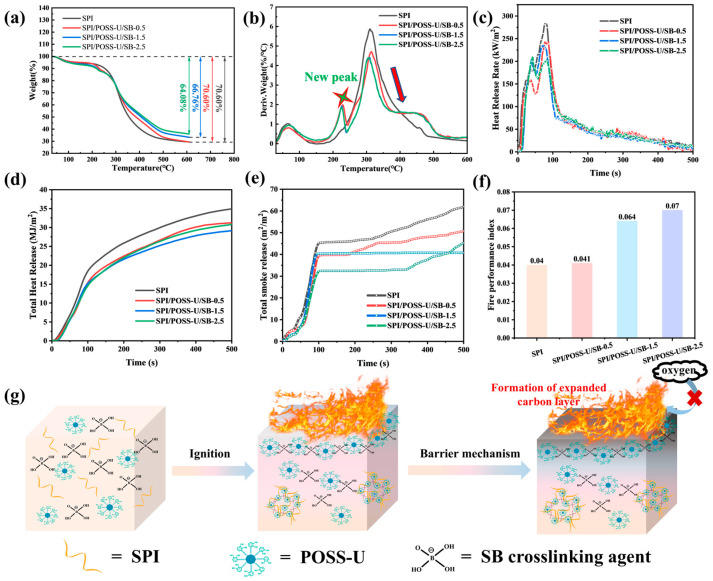
(**a**) TG curves, (**b**) DTG curves, (**c**) heat release rate, (**d**) total heat release, (**e**) total smoke release, and (**f**) fire performance index of different adhesive samples. (**g**) Flame-retardant mechanism of the SPI/POSS-U/SB adhesive.

To investigate the thermal stability of SPI adhesives before and after modification, the TGA test was conducted. From the TG curves in Figure 4a, the pristine SPI adhesive exhibited the largest weight loss of 70.60%. After introducing POSS-U and SB, the weight loss of the SPI/POSS-U/SB adhesives gradually decreased, with the SPI/POSS-U/SB-2.5 adhesive exhibiting the lowest weight loss of 64.08%. These results demonstrated that the incorporation of POSS-U/SB effectively enhanced the thermal stability of SPI adhesives. This improvement was attributed to the inherently high thermal stability of octavinyl-POSS. Moreover, the POSS-U and SB increased the crosslinking density of the SPI adhesive, which reduced the content of unreacted small molecules susceptible to thermal decomposition, thereby improving the adhesive’s thermal stability.

The DTG curves of different SPI adhesives are shown in Figure 4b. Compared to the pristine SPI adhesive, a new degradation peak appeared in the range of 200–250 °C for the SPI/POSS-U/SB adhesives. This new peak could be ascribed to the formation of new crosslinked structures facilitated by SB. Additionally, the maximum degradation rate of the SPI/POSS-U/SB adhesives in the range of 250–400 °C was significantly lower than that of the pure SPI adhesive, and the degradation rate further decreased with increasing SB content. These findings further confirmed that the SPI/POSS-U/SB adhesives exhibited enhanced thermal stability. This improvement was due to the formation of a dense crosslinked network between POSS-U and SPI facilitated by SB, which improved the adhesive’s thermal stability.

To evaluate the flame retardancy of the SPI adhesives, cone calorimeter tests were performed. As shown in Figure 4c, the peak heat release rate (PHRR) of the pristine SPI adhesive was 283.1 kW/m^2^. After incorporating POSS-U and SB, the PHRR decreased significantly, with the SPI/POSS-U/SB-2.5 adhesive showing a 25.4% reduction to 211.3 kW/m^2^. Furthermore, the total heat release (THR) (Figure 4d) and total smoke release (TSR) (Figure 4e) values for the SPI/POSS-U/SB adhesives were both lower than those of the pristine SPI adhesive. The fire performance index (FPI), shown in Figure 4f, was 0.04 for the pristine SPI adhesive, whereas the SPI/POSS-U/SB-2.5 adhesive exhibited an FPI of 0.07, representing a 41.4% improvement. These results indicated that the SPI/POSS-U/SB adhesives exhibited enhanced flame retardancy.

The improved flame-retardant properties can be attributed to the synergistic flame-retardant effect of octavinyl-POSS and SB in the SPI/POSS-U/SB adhesives. As shown in Figure 4g, on one hand, the Si-O-Si cage structure of octavinyl-POSS exhibited excellent thermal stability at high temperature, effectively suppressing the thermal degradation of SPI adhesive molecular chains. Moreover, octavinyl-POSS promoted the formation of a protective char layer, which served as a thermal and oxygen barrier, inhibiting further combustion. On the other hand, under high temperature, SB underwent dehydration to form boron oxide (B_2_O_3_), which generated a protective layer on the adhesive surface. Additionally, SB facilitated the char formation of organic components within the adhesive, reducing the release of combustible volatiles. These synergistic effects enhanced the thermal shielding and char-forming capabilities of the SPI adhesive, significantly improving its flame-retardant performance.

### 3.5. Mildew Resistance of Adhesives

**Figure 5:**
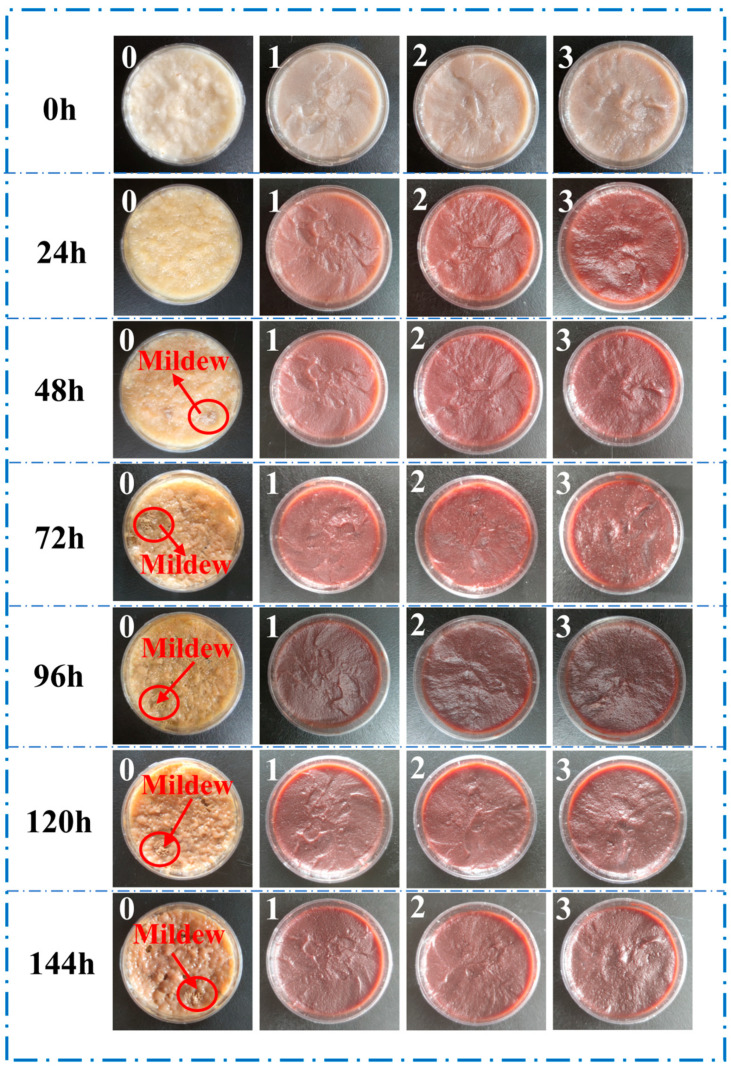
Mildew resistance measurements of different adhesives: 0-SPI, 1-SPI/POSS-U/SB-0.5, 2-SPI/POSS-U/SB-1.5, and 3-SPI/POSS-U/SB-2.5.

SPI adhesives are prone to mold growth due to their high content of nutrients, such as proteins, which are conducive to fungal proliferation. The mold resistance of adhesives directly influences their bonding performance and durability. As shown in Figure 5, the pristine SPI adhesive began to exhibit mold growth after 48 h. Over time, fungal spores proliferated extensively, leading to adhesive deterioration and the generation of an unpleasant odor. After 144 h of mildew resistance testing, the surface of the pure SPI adhesive exhibited extensive mold growth, with the proportion of the mildew area on the adhesive sample exceeding 90%, corresponding to a mildew resistance grade of 4, as shown in Table S2 (GB/T 1741-2020). Notably, after the incorporation of POSS-U and SB, no mold growth was observed on the SPI/POSS-U/SB adhesives within 0–144 h, with a mildew resistance grade of 0, demonstrating excellent antifungal properties. The enhanced mildew resistance can be attributed to the synergistic effects of urushiol (U) and SB. The polyphenolic components of U can penetrate fungal cell membranes and interact with proteins and lipids, disrupting the membrane structure. This interference inhibits the transport and metabolism of intracellular substances, ultimately leading to cell death. In addition, the boron components in SB suppress the germination and growth of fungal spores, preventing further fungal propagation. Collectively, the SPI/POSS-U/SB adhesives exhibited superior mold resistance, which significantly enhances their durability and suitability for long-term applications in humid environments.

### 3.6. Comprehensive Performance of the SPI/POSS-U/SB Adhesive

**Figure 6:**
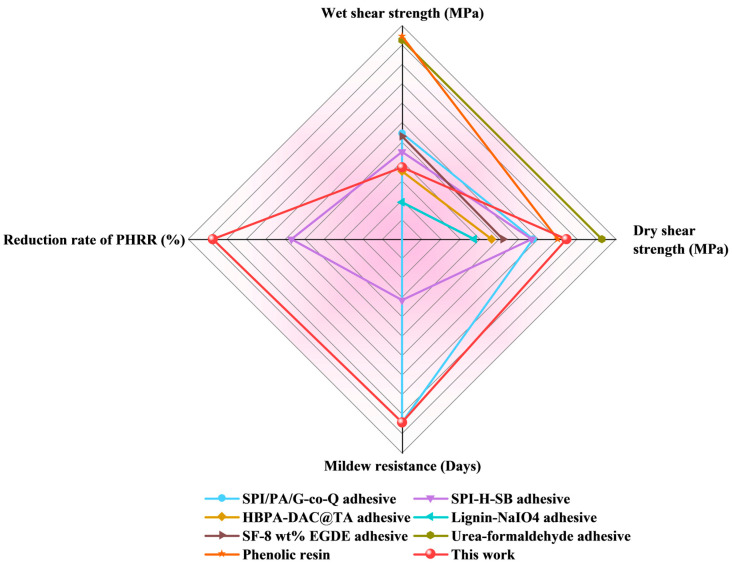
Comparison of the shear strength of SPI/POSS-U/SB with other adhesives that have been published, including the SPI/PA/G-co-Q adhesive [45], SPI-H-SB adhesive [22], HBPA-DAC@TA adhesive [46], lignin-NaIO4 adhesive [47], SF-8 wt% EGDE adhesive [48], urea-formaldehyde adhesive [49], and phenolic resin [50].

In comparison with commercially available adhesives and other bio-based alternatives reported in recent literature, the SPI/POSS-U/SB adhesive demonstrated good overall performance. As shown in Figure 6 and Table S3, the dry shear strength of the SPI/POSS-U/SB adhesive was significantly higher than that of other bio-based adhesives, even comparable to commercial adhesives, such as urea-formaldehyde and phenolic resins, highlighting its excellent bonding performance. Additionally, the wet shear strength of the SPI/POSS-U/SB adhesive met the indoor use standards (≥0.7 MPa), indicating that it exhibited sufficient water resistance. Furthermore, compared to other modification strategies for SPI-based adhesives, the SPI/POSS-U/SB adhesive exhibited outstanding flame retardancy and mildew resistance properties. Thus, the development of a high-performance, environmentally friendly, and multifunctional bio-based soy protein adhesive via core–shell hybridization and borate ester strategies offers a feasible strategy for advancing multifunctional composite materials.

## 4. Conclusions

In this study, a high-performance, eco-friendly, and multifunctional soy protein adhesive (SPI/POSS-U/SB) was successfully developed through core–shell hybridization and borate chemistry. The reactive core–shell hybrid (POSS-U), synthesized by free radical polymerization with an octavinyl-POSS core and a urushiol (U) shell, was mixed with sodium borate (SB) and SPI to prepare the SPI/POSS-U/SB adhesive. This strategy offers the following key advantages: (1) the POSS-U core–shell hybrid significantly improves the mechanical strength and toughness of the SPI adhesive. Simultaneously, the urushiol shell of the POSS-U hybrid forms hydrogen bonds and phenolic-amine crosslinked networks with SPI, further enhancing the adhesive’s bonding performance. (2) SB serves as a bridge, crosslinking hydroxyl groups in both SPI and POSS-U to construct multi-crosslinked networks, thereby improving the adhesive’s bonding performance. (3) The silicon and boron components in the SPI/POSS-U/SB adhesive provide superior flame retardancy, while the polyphenol and boron components offer excellent anti-mold properties. The SPI/POSS-U/SB-1.5 adhesive achieved a dry shear strength of 2.46 MPa and a wet shear strength of 0.74 MPa, meeting indoor application standards. Cone calorimetry showed a 25.4% reduction in the peak heat release rate (PHRR) and a 41.4% increase in the fire performance index (FPI) due to the synergistic thermal shielding and char-forming effects of octavinyl-POSS and SB. Additionally, the adhesive exhibited excellent antifungal properties, with no mold growth observed within 144 h, attributed to the polyphenolic urushiol disrupting fungal membranes and boron, inhibiting spore germination. Future research may focus on enhancing the adhesive’s toughness and durability while exploring scalable production for industrial applications in automotives, electronics, and construction. Practically, SPI/POSS-U/SB adhesives show promise in the plywood industry for indoor applications. Their sustainability and high performance make them strong candidates for large-scale adoption in eco-friendly bonding solutions.

## Figures and Tables

**Table 1:** Formulations for the preparation of adhesives.

**Sample**	**SPI (g)**	**POSS-U (g)**	**SB (g)**	**Deionized Water (g)**
0. SPI	12	0	0	88
1. SPI/POSS-U/SB-0.5	12	3	0.5	88
2. SPI/POSS-U/SB-1.5	12	3	1.5	88
3. SPI/POSS-U/SB-2.5	12	3	2.5	88

## Data Availability

The data presented in this study are available on request from the corresponding author. The data are not publicly available due to the ongoing patent application process for the technologies involved in this research, which restricts data access to protect intellectual property rights.

## Supplementary Materials

The following supporting information can be downloaded at: https://www.mdpi.com/article/10.3390/ma18051144/s1, Figure S1: The chemical structure and physical properties of octavinyl-POSS, urushiol and SPI; Figure S2: (a) the schematic of plywood under tensile shear stress, and (b) the force distribution during the test; Table S1: The evaluation criteria of mildew resistance grade; Table S2: The mildew resistance level of different adhesives after 144 h; Table S3: Comparison of the prepared SPI/POSS-U/SB adhesive and other reported adhesives from commercial and bio-based alternatives.
