# Supplementary material for: Developing Eco-Friendly, High-Performance Soy Protein Plywood Adhesive via Core–Shell Hybridization and Borate Chemistry
Source: Materials (Basel). 2025 Mar 4;18(5):1144. doi: 10.3390/ma18051144 (PMC11902272; doi:10.3390/ma18051144)
Supplement: Supplementary file 1 [file materials-18-01144-s001.zip › materials-3407728-supplementary.pdf]

Supporting Information

Developing eco-friendly, high-performance soy protein plywood adhesive via core–shell hybridization and borate chemistry

Yi Zhang[b]‡, Longxiang Sun[c]‡, Xinyu Li[b], Ziye Fu[b], Yang Li[b], Weisheng Sun[b], Yawei Sun[e], Rongfeng Huang[d,*], Minghui Guo[a,*]

[a] Key Laboratory of Bio-based Material Science & Technology, Northeast Forestry University, Ministry of Education, Harbin 150040, China

[b] College of Chemistry and Materials Engineering, Zhejiang A&F University, Hangzhou 311300, China

[c] Treessun Flooring Co., Ltd, Huzhou 313029, China

[d] Research Institute of Wood Industry, Chinese Academy of Forestry, Beijing 100091, PR China

[e] North Information Control Research Academy Group Co., Ltd, Nanjing 211100, China

‡ Y.Z. and L.S. contributed equally to this paper.

Email: huangrf@caf.ac.cn; minghuiguo202106@163.com

List of Abbreviations

U: urushiol

SB: sodium borate

SPI: soy protein isolate

APS: ammonium persulfate

NaOH: sodium hydroxide

POSS-U: reactive core–shell hybrid

POSS: polyhedral oligomeric silsesquioxane

octavinyl-POSS: octavinyl-polyhedral oligomeric silsesquioxane

1.1 Sustainability discussion

The environmental implications of raw material sourcing and production processes were discussed, with a particular focus on the synthesis of octavinyl-POSS, the extraction of urushiol (U) from raw lacquer, and the adhesive's preparation.

(1) Adhesive Raw Material Sourcing

- **Octavinyl-POSS Synthesis:** The octavinyl-POSS used in this study was commercially sourced as a pre-synthesized reagent (Aladdin Biochemical Technology Co., Ltd.). Typically, octavinyl-POSS synthesis involves the hydrolytic condensation of organosilane precursors under controlled conditions. Industrial-scale production often employs optimized, closed-loop systems to recover unreacted silane monomers and solvents, thereby reducing waste generation and mitigating environmental burdens.
- **Urushiol Extraction from Raw Lacquer:** In this study, urushiol was extracted from raw lacquer using an ethanol-based dissolution and filtration process, followed by solvent evaporation to obtain a purified product. This method eliminated the need for harsh chemicals and high-energy processing, adhering to green chemistry principles. Moreover, raw lacquer is a renewable natural resource obtained from lacquer trees, making urushiol a sustainable bio-based material.
- **Other Key Components:** Soy protein isolate (SPI), a byproduct of soybean oil processing, served as a biodegradable adhesive matrix. Sodium borate (SB), used as a cross-linker, is a low-toxicity inorganic compound that enhances adhesive performance while maintaining environmental compatibility.

(2) Production Processes

- **Synthesis of POSS-U Hybrid**: The POSS-U hybrid was synthesized via a free-radical polymerization reaction between octavinyl-POSS and urushiol, initiated by ammonium persulfate (APS). This process was conducted at ambient temperature, making it an energy-efficient and environmentally friendly approach. Additionally, APS is a relatively benign initiator that does not generate hazardous byproducts, further reducing the environmental impact compared to other polymerization methods that require toxic solvents or elevated temperatures.
- **Adhesive Preparation:** Water was used as the primary dispersion medium, eliminating emissions of volatile organic compounds (VOCs). The SPI/POSS-

U/SB adhesive was prepared via simple mixing at room temperature, ensuring a safe, low-energy, and eco-friendly fabrication process.

Overall, the SPI/POSS-U/SB adhesive system integrates bio-based and renewable materials while minimizing the use of environmentally hazardous reagents, which aligns with the principles of green chemistry and sustainable material development.

1.2 Additional Data

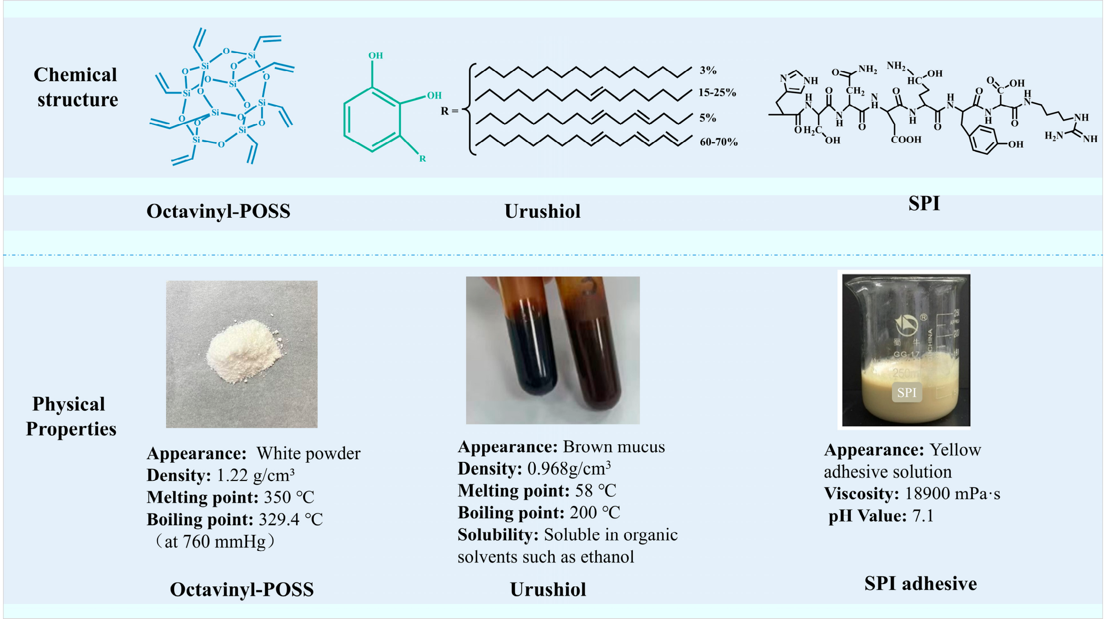

Figure S1. The chemical structure and physical properties of octavinyl-POSS, urushiol and SPI.

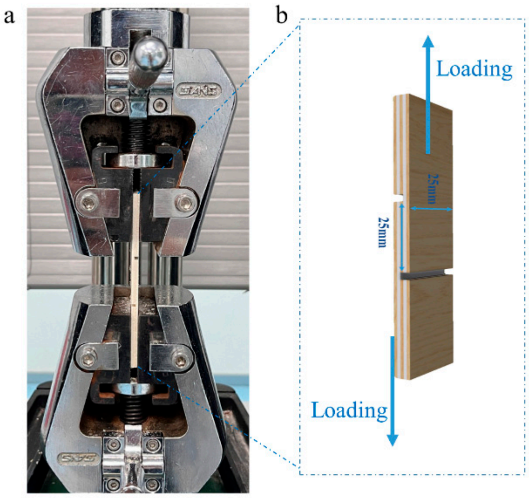

Figure S2. a) the schematic of plywood under tensile shear stress, and b) the force distribution during the test.

Table S1. The evaluation criteria of mildew resistance grade.

Mildew growth on the sample	Proportion of mildew area in the sample (%)	Mildew resistance grade
No growth	0	0
Trace Growth	<10	1
Minimal growth	10≤ area <30	2
Moderate growth	30≤ area <60	3
Severe growth	≥60	4

Table S2. The mildew resistance level of different adhesives after 144 hours.

Adhesive samples	Mildew growth on the sample	Proportion of mildew area in the sample (%)	Mildew resistance level
0-SPI	Severe growth	≥90	4
1-SPI/POSS-U/SB-0.5	No growth	0	0
2-SPI/POSS-U/SB-1.5	No growth	0	0
3-SPI/POSS-U/SB-2.5	No growth	0	0

Table S3. Comparison of the prepared SPI/POSS-U/SB adhesive and other reported adhesives from commercial and bio-based alternatives.

**Soy-based adhesive**	**Dry shear strength (MPa)**	**Wet shear strength (MPa)**	**Reduction rate of PHRR (%)**	**Mildew resistance (Days)**	**Reference**
SPI/POSS-U/SB adhesive	2.46	0.74	25.4%	6	This work
SPI/PA/G-co-Q adhesive	1.96	1.09	-	6	[45] Xu 2022
SPI-H-SB adhesive	1.94	0.90	10.7%	2	[22] Gu 2020
HBPA-DAC@TA adhesive	1.34	0.70	-	-	[46] Peng 2024
Lignin-NaIO4 adhesive	1.08	0.38	-	-	[47] Chen 2019
SF-8 wt% EGDE adhesive	1.51	1.06	-	-	[48] Yao 2019
Urea-	2.99	2.05	-	-	[49] Bekhta

formaldehyde adhesive					2021
Phenolic resin	2.33	2.09	-	-	[50] Feng 2021

References:

22. Gu, W.; Li, F.; Liu, X.; Gao, Q.; Gong, S.; Li, J.; Shi, S.Q.J.G.C. Borate chemistry inspired by cell walls converts soy protein into high-strength, antibacterial, flame-retardant adhesive. **2020**, *22*.

45. Xu, Y.; Zhang, X.; Wang, G.J.C.e.j. Preparation of a strong soy protein adhesive with mildew proof, flame-retardant, and electromagnetic shielding properties via constructing nanophase-reinforced organic-inorganic hybrid structure. **2022**, 447.

46. Peng, J.; Lei, J.; Feng, F.; Liu, F.; Ma, Y.; Bai, J.; Da, G.; Wei, C.; Huo, Z.; Cui, J. Eco-friendly and novel tannin-based wood adhesive enhanced with cellulose nanofibrils grafted by hyperbranched polyamides. *Industrial Crops and Products* **2024**, *222*, 119576, doi:https://doi.org/10.1016/j.indcrop.2024.119576.

47. Chen, X.; Xi, X.; Pizzi, C.A., Siham %J The Journal of Adhesion. Oxidized demethylated lignin as a bio-based adhesive for wood bonding. **2021**, *97*, 873-890.

48. Yao, X.; Liu, H.; Li, C.J.H. Development of Eco-Friendly Soy Meal Adhesives Enhanced by Ethylene Glycol Diglycidyl Ether. **2019**.

49. Bekhta, P.; Sedliacik, J.; Noshchenko, G.; Kacík, F.; Bekhta, N. Characteristics of beech bark and its effect on properties of UF adhesive and on bonding strength and formaldehyde emission of plywood panels. *European Journal of Wood and Wood Products* **2021**, *79*, 423-433, doi:10.1007/s00107-020-01632-8.

50. Feng, S.H.; Shui, T.; Wang, H.Y.; Ai, X.B.; Kuboki, T.; Xu, C.B.C. Properties of phenolic adhesives formulated with activated organosolv lignin derived from cornstalk. *Industrial Crops and Products* **2021**, *161*, doi:10.1016/j.indcrop.2020.113225.
